# Medial migration of a tympanostomy tube after 30 years: a rare case report

**DOI:** 10.1093/jscr/rjag436

**Published:** 2026-06-10

**Authors:** Eman Hajr

**Affiliations:** Department of Otolaryngology–Head & Neck Surgery, Imam Mohammad Ibn Saud Islamic University (IMSIU), Riyadh, Saudi Arabia

**Keywords:** tympanostomy tube, medial migration, middle ear foreign body, case report

## Abstract

Medial migration of tympanostomy tubes is a rare complication, particularly when detected decades after insertion. We report the case of a 40-year-old female with a history of bilateral tube insertion at the age of five, who presented with intermittent nasal congestion and mild allergic rhinitis. Otoscopic examination revealed an intact tympanic membrane with a bluish-green mass in the middle ear on the right side. Audiologic assessment showed normal hearing and middle ear function. The patient was asymptomatic from an otologic standpoint and opted for conservative management following counselling. This case represents the longest reported duration of medial tube migration, underscoring the potential for long-term retention without complications and the importance of individualized management in asymptomatic adults.

## Introduction

Myringotomy with ventilation tube (VT) insertion is a standard surgical procedure for the treatment of otitis media with effusion, particularly in children. Although generally safe, complications occur in up to 17% of cases and may include otorrhea, tympanosclerosis, persistent tympanic membrane perforation, granulation tissue formation, and cholesteatoma. Medial migration of the VT into the middle ear is an uncommon but documented complication, with an estimated incidence of 0.1%–1.1%.

The mechanisms of medial migration are not completely understood but may involve technical factors such as an oversized myringotomy, postoperative infection, or persistent eustachian tube dysfunction. Although several authors advocate surgical removal, especially when symptomatic, management of asymptomatic patients remains controversial.

## Case report

A 40-year-old woman presented to the otolaryngology clinic for evaluation of intermittent nasal congestion and mild allergic rhinitis. She also reported occasional bilateral ear blockage but denied hearing loss, otorrhea, tinnitus, or vertigo.

**Figure 1:**
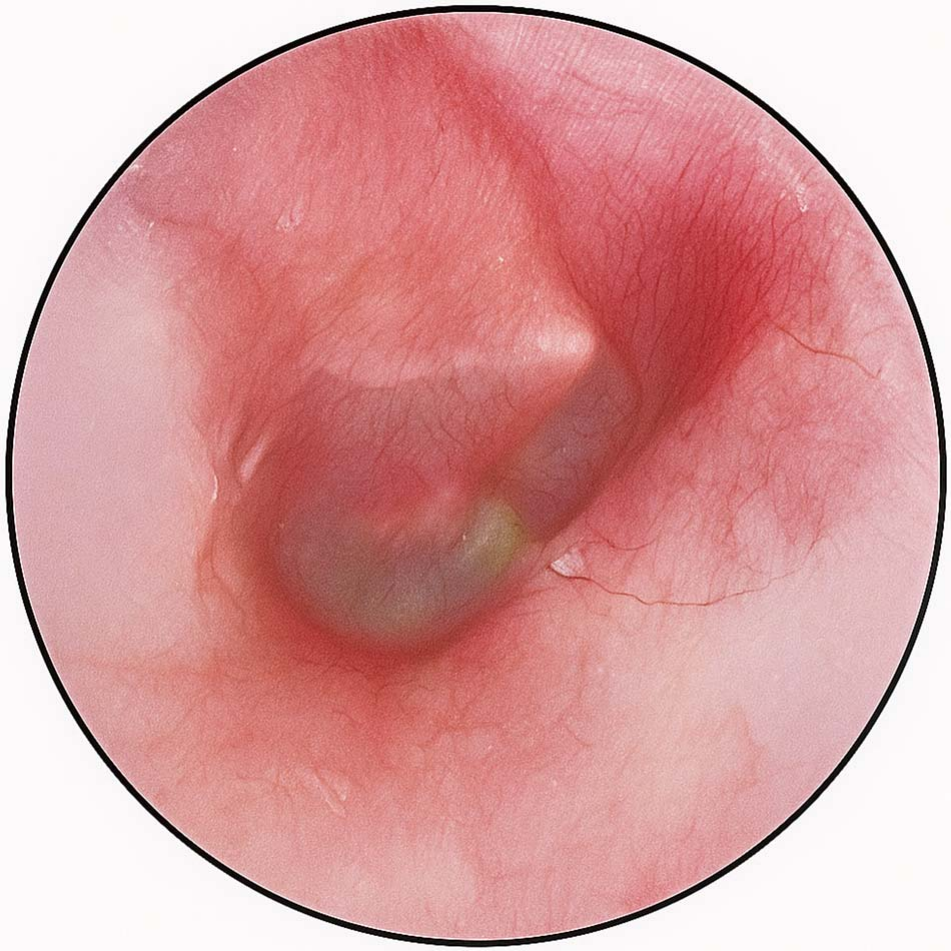
Otoscopic view of the right ear showing an intact tympanic membrane with a bluish mass in the middle ear, consistent with a medially migrated tympanostomy tube.

**Figure 2:**
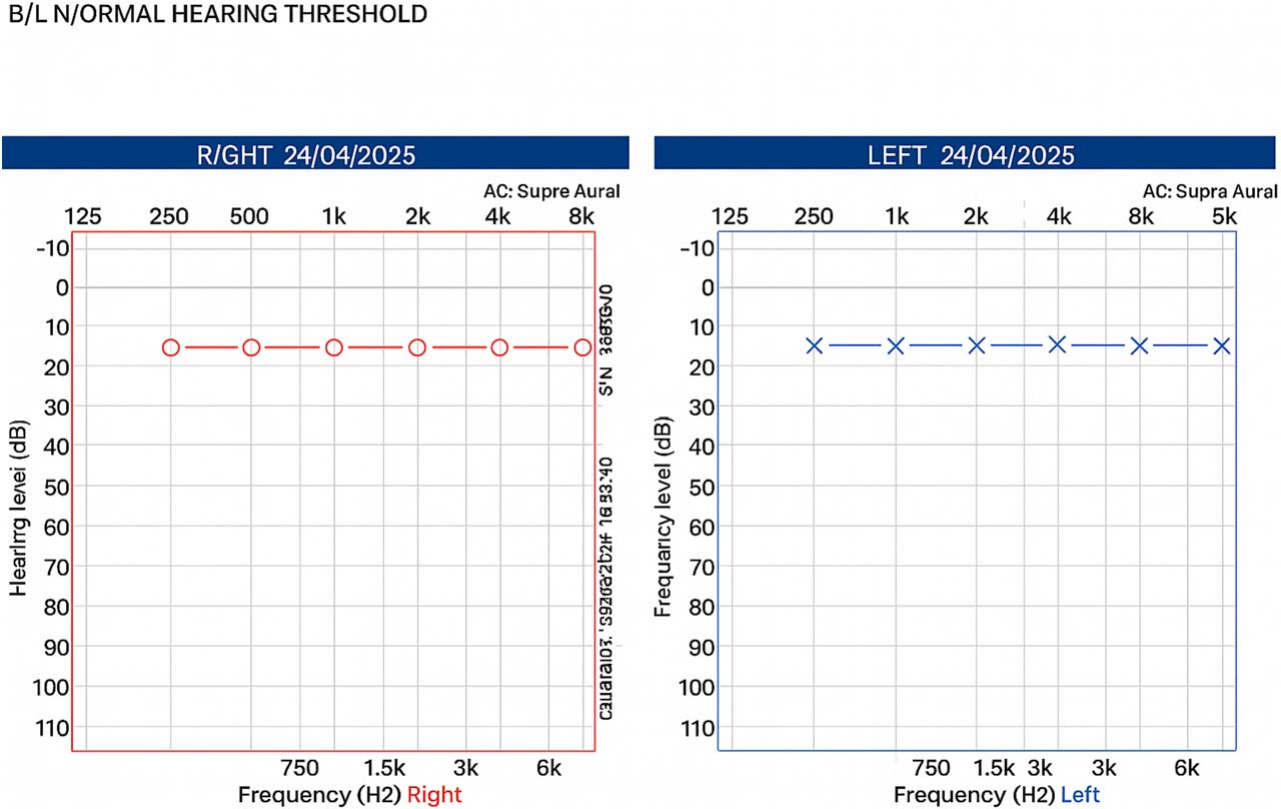
Pure tone audiometry demonstrates normal bilateral hearing thresholds.

She had a remote history of adenotonsillectomy and bilateral VT insertion at the age of five. Otoscopic examination revealed an intact tympanic membrane with a bluish-green mass visible in the middle ear on the right side (Fig. 1). The tympanic membrane demonstrated normal mobility with Valsalva manoeuvre. The left ear appeared normal. Tuning fork tests showed a centralized Weber response and positive Rinne bilaterally, indicating normal hearing. Pure tone audiometry and tympanometry confirmed normal hearing thresholds and middle ear function (Fig. 2).

When questioned, the patient denied any recent otologic procedures but recalled that her parents were informed about the prolonged presence of a tube in the right ear after surgery. She stated she had never experienced any complications from the retained tube. After counselling regarding the potential risks and benefits of removal—including possible ossicular erosion or facial weakness—she chose conservative management with periodic follow-up.

The patient remains under periodic outpatient follow-up. At the most recent review, conducted two months prior to submission, she remained asymptomatic with no evidence of otologic symptoms or audiologic deterioration.

## Discussion

Tympanostomy tube insertion is one of the most frequently performed otologic surgeries in children, typically indicated for persistent otitis media with effusion. Although generally safe, complications occur in up to 17% of cases and may include otorrhea, tympanosclerosis, persistent perforation, granulation tissue, and cholesteatoma formation [1, 2].

Medial migration of a tympanostomy tube—defined as displacement into the middle ear rather than natural extrusion into the external auditory canal—is a rare but acknowledged complication, with a reported incidence of 0.1%–1.1% [3]. The underlying mechanisms include technical factors such as an oversized or improperly placed myringotomy, postoperative infection leading to retraction, persistent negative middle ear pressure from eustachian tube dysfunction, or blockage of the tube lumen [4–6].

Clinically, medial migration may be asymptomatic and discovered incidentally or present with ear fullness, conductive hearing loss, chronic otorrhea, or complications such as ossicular damage, cholesteatoma, or perilymphatic fistula [7, 8]. Bezdjian *et al*. [9] reviewed multiple cases and found nearly half were asymptomatic at diagnosis.

While some authors advocate conservative monitoring in asymptomatic cases [10], others recommend surgical removal due to potential long-term risks [1]. Our case represents an exceptionally rare instance of a tympanostomy tube retained for over 35 years without audiologic or structural sequelae—the longest reported duration to date.

This prolonged retention without adverse outcomes reinforces the importance of individualized management and shared decision-making. After counselling about potential risks—including ossicular erosion, infection, and cholesteatoma—the patient opted for observation, which remains appropriate given her stable findings and lack of symptoms.

To our knowledge, this represents the longest reported duration of tympanostomy tube retention, illustrating that inert materials may remain quiescent in the middle ear for decades without adverse effects. This supports a conservative approach in select asymptomatic adults and highlights the need for long-term documentation following paediatric VT insertion.

## Conclusion

Medial migration of tympanostomy tubes is an uncommon but important clinical finding. This case demonstrates that conservative management can be a reasonable and safe option for asymptomatic patients, even when the tube has remained in place for decades. Shared decision-making and long-term follow-up are essential to ensure continued safety and patient satisfaction.

## Data Availability

The data supporting this case report are available from the corresponding author upon reasonable request. Due to patient confidentiality, not all data can be shared publicly.
